# Myeloid DRP1 Sulfenylation Drives Reparative Macrophage Polarization and Neovascularization in Ischemic Muscle

**DOI:** 10.3390/antiox15060768

**Published:** 2026-06-19

**Authors:** Shikha Yadav, Rajagopal Kamarajan, Varadarajan Sudhahar, Sheela Nagarkoti, Archita Das, Stephanie Kelley Spears, Rajalakshmi Veeranan Karmegam, Tohru Fukai, Masuko Ushio-Fukai

**Affiliations:** 1 Vascular Biology Center, Medical College of Georgia, Augusta University, Augusta, GA 30912, USAtfukai@augusta.edu (T.F.); 2 Department of Pharmacology and Toxicology, Medical College of Georgia, Augusta University, Augusta, GA 30912, USA; 3 Charlie Norwood Veterans Affairs Medical Center, Augusta, GA 30912, USA; 4 Department of Medicine (Cardiology), Medical College of Georgia, Augusta University, Augusta, GA 30912, USA

**Keywords:** DRP1, post-translational modification, sulfenylation, mitochondrial fission, hindlimb ischemia, peripheral arterial disease, macrophage, inflammation, metabolic reprogramming

## Abstract

Reparative macrophage polarization and macrophage-derived reactive oxygen species (ROS) are required for ischemia-induced revascularization in peripheral artery disease (PAD). Our previous study showed that mitochondrial fission protein dynamin-related protein 1 (DRP1) promotes reparative polarization and metabolic reprogramming in macrophages and post-ischemic neovascularization. However, the redox-dependent mechanism governing DRP1 activation in this context remains elusive. Here, using a mouse hindlimb ischemia (HLI) model of PAD, we identify cysteine sulfenylation (CysOH) of DRP1 as a critical redox modification induced in ischemic bone marrow (BM)-derived cells. BM chimeric mice reconstituted with CRISPR/Cas9-generated “redox-dead” DRP1-C631A knock-in mutant (*Drp1^C/A^*) BM exhibited markedly reduced limb perfusion recovery and CD31^+^ capillary density in ischemic muscles following HLI. These defects were associated with enhanced Ly6G^+^ neutrophil accumulation, pro-inflammatory F4/80^+^CD80^+^ M1-like macrophages and reduced anti-inflammatory F4/80^+^CD206^+^ M2-like macrophages in ischemic muscle. Mechanistically, using an in vitro PAD model, hypoxia serum starvation (HSS) rapidly induced NADPH oxidase 2-dependent cytosolic ROS production and DRP1-CysOH formation in wild-type macrophages. In contrast, *Drp1^C/A^* macrophages failed to undergo DRP1-CysOH-dependent mitochondrial fission under HSS, resulting in aberrant metabolic reprogramming characterized by enhanced glycolysis and mitochondrial ROS, pro-inflammatory p-NF-κB and M1-genes, and suppressed anti-inflammatory p-AMPK, efferocytosis and M2-genes. Thus, our findings establish DRP1 sulfenylation as a previously unrecognized redox-sensing mechanism that links ischemia-induced ROS to reparative macrophage reprogramming and revascularization, identifying a novel therapeutic target for PAD.

## 1. Introduction

Peripheral artery disease (PAD) is a common aging-related cardiovascular disorder characterized by impaired ischemia-induced revascularization, chronic inflammation, and progressive tissue damage that can culminate in limb loss [1]. Despite advances in revascularization procedures, there are no effective therapies that restore endogenous vascular regeneration in patients with critical limb ischemia. A major barrier to therapeutic development is the incomplete understanding of how inflammatory and reparative immune responses are regulated within ischemic tissue [1,2,3,4]. Monocyte/macrophage recruitment, efferocytosis, the clearance of apoptotic cells by macrophage, macrophage polarization, and metabolic reprogramming represent critical therapeutic targets for resolving vascular inflammation and enabling revascularization of ischemic muscle [4]. Macrophage phenotypic switching from pro-inflammatory M1-like macrophages that amplify tissue injury and recruit neutrophils to reparative M2-like macrophages that promote angiogenesis is essential for post-ischemic vascular regeneration [4,5]. The balance between these macrophage states determines whether ischemic tissue undergoes enhanced or impaired regeneration. However, the molecular mechanisms that instruct macrophage fate decisions in response to ischemia remain incompletely defined.

Emerging evidence suggests that reactive oxygen species (ROS) derived from NADPH oxidase (NOX) at optimal levels function as signaling molecules to promote ischemia-induced reparative revascularization [6,7,8,9,10]. We reported that NOX2-derived ROS in bone marrow (BM) cells and infiltrating immune cells act as key regulators of regenerative myelopoiesis and reparative neovascularization following hindlimb ischemia (HLI), a preclinical model of PAD [11,12].

However, whether and how ROS-mediated redox signaling regulates macrophage reprogramming during ischemia-driven neovascularization remains unknown. ROS activates redox signaling mainly through oxidative modification of redox-sensitive cysteine residues, generating cysteine sulfenic acid (Cys-OH), a transient intermediate that can drive disulfide bond formation and alter protein conformation, activity, and downstream signaling [13,14,15].

Mitochondria are central regulators of macrophage activation, metabolism, and survival, thereby shaping immune responses. Changes in mitochondrial respiration, mitochondrial ROS (mitoROS), tricarboxylic acid (TCA) cycle metabolites, membrane potential, and morphology influence macrophage polarization toward distinct functional states [16]. Pro-inflammatory M1-like macrophages are characterized by enhanced glycolysis, NF-κB activation, and impaired oxidative phosphorylation, whereas anti-inflammatory and pro-angiogenic M2-like macrophages depend on intact mitochondrial respiration and AMP-activated protein kinase (AMPK) signaling, which counteracts NF-κB-driven inflammatory responses [16,17,18,19]. The mitochondrial fission GTPase dynamin-related protein 1 (DRP1) regulates macrophage reprogramming in a context-dependent manner and its activity is controlled by multiple post-translational modifications such as phosphorylation, sumoylation, ubiquitination, and S-nitrosylation [20,21,22,23]. We previously demonstrated that myeloid-specific DRP1 deficiency impairs reparative macrophage polarization, metabolic reprogramming, and post-ischemic neovascularization [24]. Importantly, ischemia-induced DRP1 activation occurred independently of canonical phosphorylation at Ser616 or Ser637, suggesting a non-canonical regulatory mechanism. Because DRP1 contains a redox-sensitive cysteine residue within its GTPase effector domain (Cys644 in human; Cys631 in mouse), we hypothesized that ischemia activates DRP1 in macrophages through a redox-dependent cysteine oxidation, thereby promoting reparative macrophage polarization and neovascularization.

In this study, using a mouse HLI model of PAD and bone marrow (BM) chimeras reconstituted with CRISPR/Cas9-generated “redox-dead” DRP1^C631A^ knock-in mutant (*Drp1^C/A^*) mice [25], we identify NOX2-dependent DRP1 sulfenylation at Cys^631^ in macrophages as a critical redox switch that links ischemia-induced ROS to drive reparative macrophage reprograming and revascularization. Mechanistically, loss of DRP1 sulfenylation in macrophages failed to undergo proper DRP1-dependent fission and metabolic reprogramming characterized by excessive mitoROS production, glycolytic flux, pro-inflammatory p-NfkB and suppressed anti-inflammatory p-AMPK and efferocytosis, thereby skewing toward a pro-inflammatory M1-like macrophage phenotype, which contributes to impairing reparative neovascularization.

## 2. Materials and Methods

### 2.1. Ethics Statement for Animal Study

All animal studies were conducted in accordance with protocols approved by the Augusta University Institutional Animal Care and Use Committee and Institutional Biosafety Committee. Animals were housed in ventilated cages (2–5 mice per cage) under controlled environmental conditions (22.5 °C; 50–60% humidity) with a 12 h light/dark cycle. A CRISPR/Cas9-generated knock-in mouse carrying a cysteine-to-alanine substitution at the conserved DRP1 redox-sensitive residue (mouse Cys631, corresponding to human Cys644) was produced by the Augusta University Transgenic and Genomic Core Facility. Genotype verification was performed by PCR and targeted next-generation sequencing as previously reported [26]. Correct targeting of the DRP1 locus was confirmed by PCR and next-generation amplicon sequencing, as reported [26]. Homozygous *Drp1^C/A^* KI mice were viable, fertile, and exhibited body weights comparable to C57BL/6 wild-type (WT) mice. Age- and sex-matched WT and *Drp1^C/A^* KI mice (12–18 weeks old) were used for all in vivo and ex vivo BMDM culture experiments.

### 2.2. Hindlimb Ischemia Model

Experimental hindlimb ischemia was induced in WT and Drp1-C^631^A (Drp1-C/A) mice under ketamine/xylazine anesthesia (87/13 mg/kg, intraperitoneal). Following exposure of the femoral artery, proximal and distal ligations were placed using 6–0 silk sutures, and the intervening arterial segment was removed while preserving the adjacent nerve. The incision was closed with nylon sutures. Limb perfusion was monitored using laser Doppler perfusion imaging (PeriScan PIM 3,Perimed AB Stockholm, Sweden) [6,11]. Mice were anesthetized and placed on a heating plate at 37 °C for 10 min to minimize temperature variation. Before and after surgery, LDBF analysis was performed in the plantar sole. Blood flow recovery was calculated as the ratio of perfusion in the ischemic limb to that of the contralateral control limb.

### 2.3. BM Transplantation (BMT)

BMT was performed as described previously [11,15,26]. Donor marrow cells were isolated and purified before intravenous delivery into lethally irradiated recipient mice (9.5 Gy). Each recipient received 3 × 10^6^ donor cells 24 h after irradiation. Reconstitution was allowed for 4–6 weeks before induction of hindlimb ischemia.

### 2.4. Histological and Western Blot Analysis

For tissue analysis, animals were perfused with saline through the left ventricle prior to tissue collection. Gastrocnemius muscles were fixed in 4% paraformaldehyde, cryoprotected in sucrose, embedded in OCT, and sectioned at 7 μm thickness. Endothelial cells, macrophages, and neutrophils were identified using antibodies against CD31, F4/80, and Ly6G, respectively. Immunohistochemical detection was performed using a peroxidase-based staining system followed by DAB development. Images were captured using Keyence flourscecne miscroscope BZ-X710 (Osaka, Japan) or Zeiss 780 inverted confocal microscope (AG, Jena, Germany) and quantified using ImageJ 1.49v or Zeiss analysis software (ZEN Version 3.12.107.0300). Protein expression was evaluated by immunoblotting using standard procedures as previously described [11,15,26].

### 2.5. Flow Cytometry (FACS) Analysis

Peripheral blood was collected by cardiac puncture into EDTA-coated syringes, while bone marrow cells were obtained from femurs and tibias. Following collection, erythrocytes were removed using RBC lysis buffer (Invitrogen, Thermo Fisher Scientific, Boston, MA, USA), and the remaining leukocytes were washed and maintained in ice-cold DPBS containing 2% FCS. For analysis of ischemic tissues, gastrocnemius muscles were excised, finely minced, and digested for 1 h at 37 °C in an enzyme cocktail containing collagenase I, collagenase XI, hyaluronidase, and DNase I. The resulting cell suspension was sequentially filtered through 70 and 40 μm strainers and enumerated before staining. To minimize nonspecific antibody binding, cells were incubated with Fc receptor-blocking reagent (anti-CD16/CD32) in the presence of 2% FCS. Samples were subsequently labeled with fluorophore-conjugated antibodies against CD45, CD11b, Ly6G, Ly6C, F4/80, CD206, and CD80, together with a viability dye. Antibody information is provided in Supplementary Table S1. Corresponding isotype controls were included to establish gating parameters. After fixation with 4% paraformaldehyde, samples were analyzed using an Attune NxT flow cytometer (Thermo Fisher Scientific, Boston, MA, USA), and data were processed with FlowJo v10 software, as we reported [24].

### 2.6. Isolation and Primary Culture of BM-Derived Macrophages (BMDMs)

Primary macrophages were generated from bone marrow harvested from age-matched WT and *Drp1^C/A^* mice. Bone marrow cells were flushed from femurs and tibias, passed through a 70 μm cell strainer, and cultured in DMEM supplemented with 10% fetal bovine serum, antibiotics, and 20% L929 cell-conditioned medium as a source of macrophage colony-stimulating factors. Cells were maintained for seven days with periodic removal of non-adherent cells. Differentiation into macrophages was confirmed by flow cytometric analysis of CD11b, Ly6C, and F4/80 expression using antibodies listed in Supplementary Table S2.

### 2.7. In Vitro Hypoxia Serum Starvation (HSS)

To mimic ischemic stress in vitro, differentiated BMDMs were rinsed to remove residual serum and transferred to serum-free starvation medium (Cell Applications Inc., San Diego, CA, USA). Cells were then exposed to a hypoxic environment (2% O_2_) for the indicated time periods, as described [24].

### 2.8. Quantitative RT-PCR

Total RNA was isolated from cultured cells or tissue samples using TRI Reagent followed by phenol–chloroform extraction. Complementary DNA was synthesized from 2 μg RNA using a high-capacity reverse transcription kit (Applied Biosystems, Thermo Fisher Scientific, Boston, MA, USA). Quantitative PCR was performed with SYBR Green chemistry (Qiagen, Hilden, Germany) on an ABI Prism 7000 detection platform according to the manufacturers’ instructions. Primer sequences are listed in Supplementary Table S2. Relative transcript abundance was calculated after normalization to HPRT expression.

### 2.9. Detection of Protein Sulfenylation by DCP-Bio1 Labeling

Protein cysteine sulfenic acid formation was assessed using the sulfenic acid-reactive probe DCP-Bio1. Tissues or cells were lysed under degassed conditions in buffer containing HEPES, EDTA, NaCl, phosphatase inhibitors, iodoacetamide, DTPA, Triton X-100, catalase, protease inhibitors, and DCP-Bio1. Biotinylated proteins generated through DCP-Bio1 labeling were captured with streptavidin-conjugated beads overnight at 4 °C. Following enrichment, sulfenylated proteins were detected by immunoblotting with the indicated antibodies. To evaluate assay specificity, parallel negative-control samples were processed using streptavidin beads incubated with DCP-Bio1-containing lysis buffer in the absence of biological samples. The absence of signal in these controls confirmed minimal nonspecific binding attributable to the beads or assay reagents, as reported [26]. As negative control, streptavidin beads incubated with lysis buffer containing DCP-Bio1 in the absence of cell lysates were used [15,26].

### 2.10. BMDM Efferocytosis Assay

THP-1 monocytes in RPMI supplemented with 10% FCS were fluorescently labeled by incubating with 5 µM of CellTracker^TM^ Green (CMFDA) (Invitrogen, C7025) for 20 min. Labeled apoptotic THP-1 cells were generated by exposing (10 million cells per mL in a 10 mm cell culture dish) overnight in 1 µM Staurosporine (81590-1, Cayman chemical, Ann Arbor, MI, USA)) at 37 degrees Celsius. Late apoptotic or necrotic THP-1 cells were generated by incubation at 56 degrees Celsius for 30 min. A mixture of early apoptotic and late apoptotic (10:1) cells were used for the efferocytosis assay. BMDMs with or without hypoxia serum starvation (HSS) for 8 h were incubated with 5:1 CMFDA^+^ early and late apoptotic monocytes cells for 2 h under normoxia or HSS conditions. BMDMs were washed three times with 1X PBS to remove unbound cells and then harvested in Trizol for RT-PCR analysis of efferocytotic marker genes or immunostained with PE-anti-mouse F4/80 (clone BM8) (BioLegend, San Diego, CA, USA, 23110) and assessed for apoptotic cell uptake by IF imaging as reported [27,28].

### 2.11. BMDM Mitochondrial Structure, Mitochondrial Potential and mtROS Analysis

BMDMs under normoxia or HSS stimulation were stained as per the manufacturer’s protocol with MitoTracker Red FM (M22425, Invitrogen) at 100 nM, mitoSOX TM Red (M36008, Invitrogen) at 5 µM or Image-iT^TM^ TMRM (I34361, Invitrogen) at 200 nM to visualize mitochondrial structure, mtROS and mitochondrial membrane potential respectively. Images were acquired by confocal microscopy (Zeiss 780 inverted confocal microscope (AG, Jena, Germany) or Nikon AX NSPARC, (Nikon Corporation, Tokyo, Japan) and fluorescence intensity was quantified using ImageJ software.

### 2.12. BMDM CellROX Measurement

BMDMs under normoxia and HSS stimulation with or without specific small-molecule NOX2 inhibitor (GSK2795039, CAYMAN) pre-treatment at 10 µM for 2 h as reported [29] were stained with CellROX^TM^ Orange Reagent (C10443, Invitrogen) for 30 min at 5 µM using the manufacturer’s protocol as reported [26]. CellROX fluorescence was imaged by Zeiss LSM 710 confocal microscopy. Total cellular ROS intensity was quantified using ImageJ software.

### 2.13. Statistical Analysis

All experiments were performed a minimum of 3 independent times, and results are expressed as mean ± SEM. Differences between two groups were evaluated using an unpaired two-tailed Student’s *t*-test. Comparisons involving more than two groups were analyzed by one-way ANOVA followed by Tukey’s post hoc test or Bonferroni correction for multiple comparisons, as appropriate. Values of * *p* < 0.05, ** *p* < 0.01, and *** *p* < 0.001 were considered statistically significant. Statistical analyses were conducted using GraphPad Prism version 10 (GraphPad Software10.6.1San Diego, CA, USA).

## 3. Results

### 3.1. Myeloid DRP1 Sulfenylation Is Required for Ischemia-Induced Reparative Angiogenesis in Response to HLI

**Figure 1:**
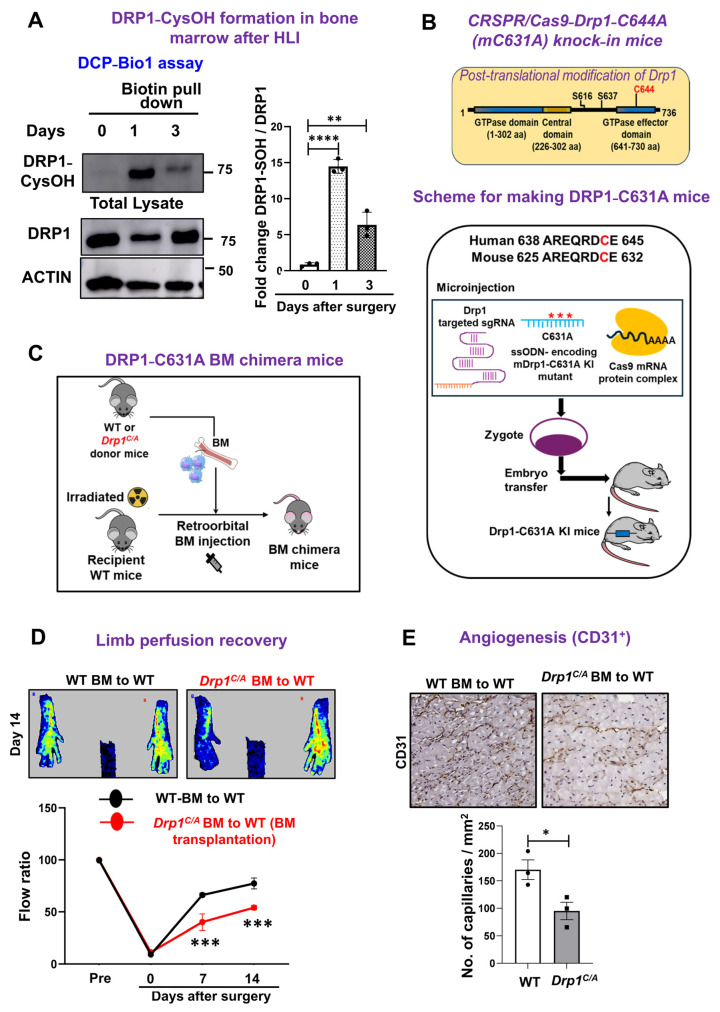
***Drp1^C/A^*** **bone marrow chimera mice exhibit impaired revascularization after HLI.** (**A**) Immunobloting for DRP1Cys-OH in ischemic and non-ischemic bone marrow (BM) of WT mice at indicated days after HLI measured by DCP-Bio1 assay. DCP-Bio1 pulldown experiments were performed with a “beads-only” negative control consisting of streptavidin beads incubated with lysis buffer containing DCP-Bio1 in the absence of cell lysates. n = 3 sets per group pooled from 2 WT bone marrow, 1-way ANOVA followed by Tukey’s multiple comparisons test. (**B**) Schematic of CRISPR/CAS9-mediated “redox-dead” mDRP1-C631A (corresponding to human DRP1-Cys644A) knock-in mutant mice generation. (**C**) Schematic of WT and *Drp1^C/A^* bone marrow transplantation (BMT) and hindlimb ischemia. (**D**) Blood flow recovery (ischemic/non-ischemic legs) of WT (black) and *Drp1^C/A^* (red) after HLI measured by laser Doppler flow analyzer n= 4 mice per group, 2-way ANOVA followed by Bonferroni’s multiple comparisons test. (**E**) CD31^+^ angiogenesis in GC muscle at day 21 after HLI. Scale bar: 20 µM. n = 3 mice per group, unpaired Student’s *t*-test. Sex- and age-matched 12–18-week-old WT and *Drp1^C/A^* mice were used. Data are mean ± SEM (n = 3–4). * *p* < 0.05, ** *p* < 0.01, *** *p* < 0.001, **** *p* < 0.0001.

We previously demonstrated that myeloid DRP1 and BM-derived ROS are essential for post-ischemic reparative angiogenesis in the HLI model of PAD [11,24,30,31]. Notably, ischemia activates DRP1-dependent mitochondrial fission in macrophages without increasing canonical DRP1 phosphorylation, suggesting a non-phosphorylation-based activation mechanism. We therefore investigated whether ischemia induces cysteine oxidation of DRP1 in vivo and whether this modification is functionally required for reparative neovascularization. Using the HLI model, DCP-Bio1 assay revealed that DRP1 sulfenylation (DRP1-CysOH) in BM cells was markedly increased at day 1 post HLI compared with non-ischemic BM cells, and gradually declined by day 3 (Figure 1A). To define the functional significance of endogenous DRP1 cysteine oxidation, we generated a CRISPR/Cas9-engineered “redox-dead” Drp1^C631A^ (*Drp1^C/A^*) KI mice (Figure 1B), as described [25]. To specifically assess the role of myeloid DRP1 sulfenylation in post-ischemic angiogenesis, we performed BM transplantation (BMT) to reconstitute lethally irradiated WT recipients with BM from either WT or *Drp1^C/A^* KI donors (Figure 1C). Laser speckle contrast imaging revealed that perfusion recovery at days 7 and 14 following HLI was significantly impaired in *Drp1^C/A^*-to-WT BM chimeric mice compared with WT-to-WT controls (Figure 1D). Consistent with these findings, immunohistochemical analysis of ischemic muscle demonstrated a marked reduction in CD31-positive capillary density in *Drp1^C/A^* -to-WT mice after HLI (Figure 1E). Collectively, these results demonstrate that myeloid DRP1 sulfenylation is required for effective perfusion recovery and reparative neovascularization following ischemic injury, identifying DRP1 cysteine oxidation as a critical redox-dependent mechanism linking ischemia to macrophage-driven angiogenesis.

### 3.2. Myeloid DRP1 Sulfenylation Promotes Macrophage Efferocytosis and Pro-Angiogenic M2-like Macrophage Polarization in Ischemic Muscle

**Figure 2:**
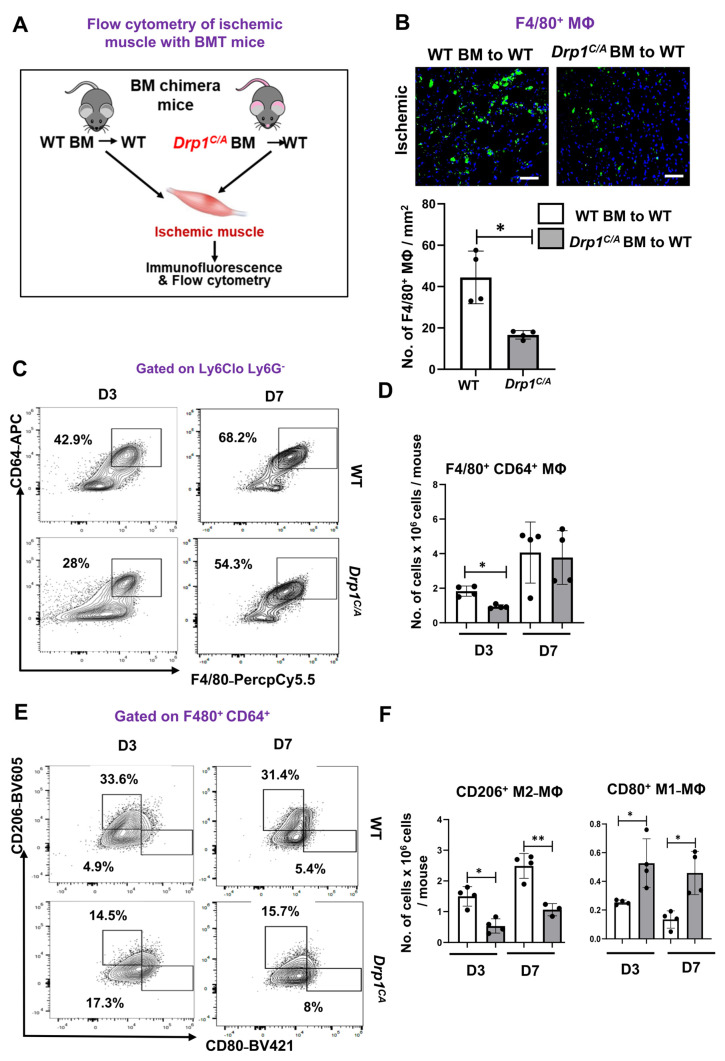
***Drp1 ^C/A^*** **bone marrow chimera mice show increased pro-inflammatory M1-MΦ polarization and decreased anti-inflammatory M2-MΦ polarization in ischemic muscles after HLI.** (**A**) Schematic of flow cytometry-based immunophenotyping of ischemic gastrocnemius (GC) muscle after BMT and HLI surgery. (**B**) Representative immunofluorescence images of F4/80^+^ (green) macrophages and quantification in GC muscle at day 3 after HLI. Scale bar: 20 µM. n = 4 mice per group, unpaired Student’s *t*-test. (**C**,**D**) Representative FACS contour plot of total F4/80^+^ CD64^+^ macrophages and quantification. n = 4 mice per group, 1-way ANOVA followed by Bonferroni’s multiple comparisons test. (**E**,**F**) CD80^+^M1- and CD206^+^M2-MΦ and quantification in ischemic GC muscle at day 3 and day 7 post HLI. n = 4 mice per group, 1-way ANOVA followed by Bonferroni’s multiple comparisons test. Sex- and age-matched 12–18-week-old WT and *Drp1^C/A^* mice were used. Data are mean ± SEM (n = 4 mice). * *p* < 0.05, ** *p* < 0.01.

**Figure 3:**
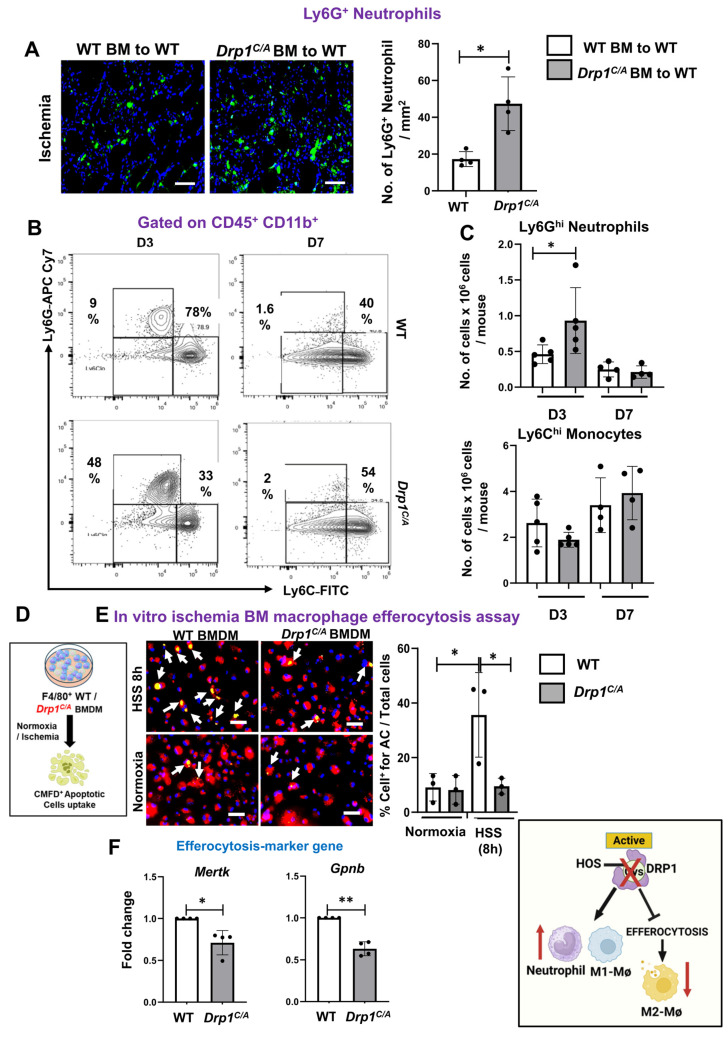
**Increased neutrophils in ischemic muscle and impaired efferocytosis in** ***Drp1^C/A^*** **BMDMs during** ***in vitro*** **ischemia.** (**A**) Representative immunofluorescence images of Ly6G^+^ neutrophils (green) and quantification in GC muscle at day 3 after HLI. Scale bar: 20 µM. n = 4 mice per group, unpaired Student’s *t*-test. (**B**,**C**) Representative flow cytometry contour plots and quantification of numbers of Ly6G^hi^ neutrophils and Ly6C^hi^ monocytes in ischemic GC muscle of WT and *Drp1^C/A^* mice after day 3 of HLI. n = 4 mice per group, 1-way ANOVA followed by Bonferroni’s multiple comparisons test. (**D**) Schematic representation of in vitro BMDM efferocytosis assay. (**E**) IF imaging and quantification of labeled apoptotic cell uptake in WT and *Drp1^C/A^* BMDMs during normoxia and 8 h of HSS stimulation (indicated by arrow head)Scale bar: 20 µM. n = 3 mice per group, 1-way ANOVA followed by Bonferroni’s multiple comparisons test. (**F**) qRT-PCR analysis of *Mertk* and *Gpnb* gene normalized to *Hprt* in WT and *Drp1^C/A^* BMDMs after 8 h of HSS and treatment with apoptotic cells for 2 h. n = 4 sets of BMDMs per group. BMDMs from sex- and age-matched 12–18-week-old WT and *Drp1^C/A^* mice were used. Data are mean ± SEM. * *p* < 0.05, ** *p* < 0.01 (Schematic hypothesis model, created in BioRender. Ushio-Fukai, M. (2026), https://BioRender.com/96r8gxt).

To determine whether myeloid DRP1 sulfenylation regulates immune cell infiltration, macrophage differentiation, and polarization during ischemic injury, we performed flow cytometry-based immunophenotyping of ischemic GC muscles from WT and *Drp1^C/A^* KI BM chimera mice at days 3 and 7 following HLI (Figure 2A). Immunofluorescence analysis revealed a decrease in total number of F4/80^+^ macrophages in GC muscle of *Drp1^C/A^* KI BM chimera compared with WT mice (Figure 2B). Further, flow cytometry analysis confirmed that the number of F4/80^+^CD64^+^ mature macrophages was significantly reduced at day 3, but not at day 7, after HLI in *Drp1^C/A^* KI BM chimeric mice compared with WT controls (Figure 2C,D). Notably, inhibition of myeloid DRP1 cysteine oxidation resulted in a marked shift in macrophage polarization. The number of F4/80^+^CD64^+^CD206^+^ M2-like pro-angiogenic macrophages significantly decreased, whereas F4/80^+^CD64^+^CD80^+^ M1-like pro-inflammatory macrophages significantly increased at both days 3 and 7 after HLI in ischemic muscle of *Drp1^C/A^* KI BM chimera mice relative to WT mice (Figure 2E,F). We next assessed whether myeloid DRP1-CysOH reduction alters monocyte or neutrophil recruitment to ischemic tissue. While Ly6G^+^ neutrophils were significantly increased at day 3 after HLI in *Drp1^C/A^* KI BM chimera mice compared with WT mice; their numbers declined by day 7 (Figure 3A–C). In contrast, Ly6Chi monocyte numbers were comparable between genotypes at both days 3 and 7 following HLI (Figure 3B,C).

Given the critical role of efferocytosis in resolving inflammation and promoting reparative M2 macrophage polarization, we performed in vitro efferocytosis assays [28,32] using WT and *Drp1^C/A^* BMDMs under basal and HSS conditions. HSS stimulation for 8 h significantly enhanced the uptake of apoptotic cells by WT BMDMs compared with normoxic controls. In contrast, *Drp1^C/A^* BMDMs exhibited significantly impaired efferocytosis following HSS stimulation compared with WT BMDMs, whereas no difference was observed under normoxic conditions (Figure 3D,E). Consistent with these findings, the HSS-induced expression of efferocytosis-associated genes (*Mertk* and *Gpnb*) was markedly reduced in *Drp1^C/A^* BMDMs relative to WT BMDMs (Figure 3F). Together, these findings demonstrate that myeloid DRP1 sulfenylation is not required for monocyte recruitment but is essential for macrophage reprogramming toward a reparative, pro-angiogenic M2-like phenotype in ischemic muscle. Loss of DRP1-CysOH shifts macrophages toward a pro-inflammatory M1-like state, accompanied by impaired efferocytosis and increased neutrophil accumulation. These alterations likely contribute to unresolved inflammation and establish a mechanistic link between defective macrophage reprogramming and impaired ischemia-induced neovascularization following HLI.

### 3.3. Inhibition of Ischemia-Induced DRP1 Sulfenylation Promotes Mitochondrial Hyperfusion, Reduced Membrane Potential, and Excessive mitoROS Production in Macrophages in Vitro

**Figure 4:**
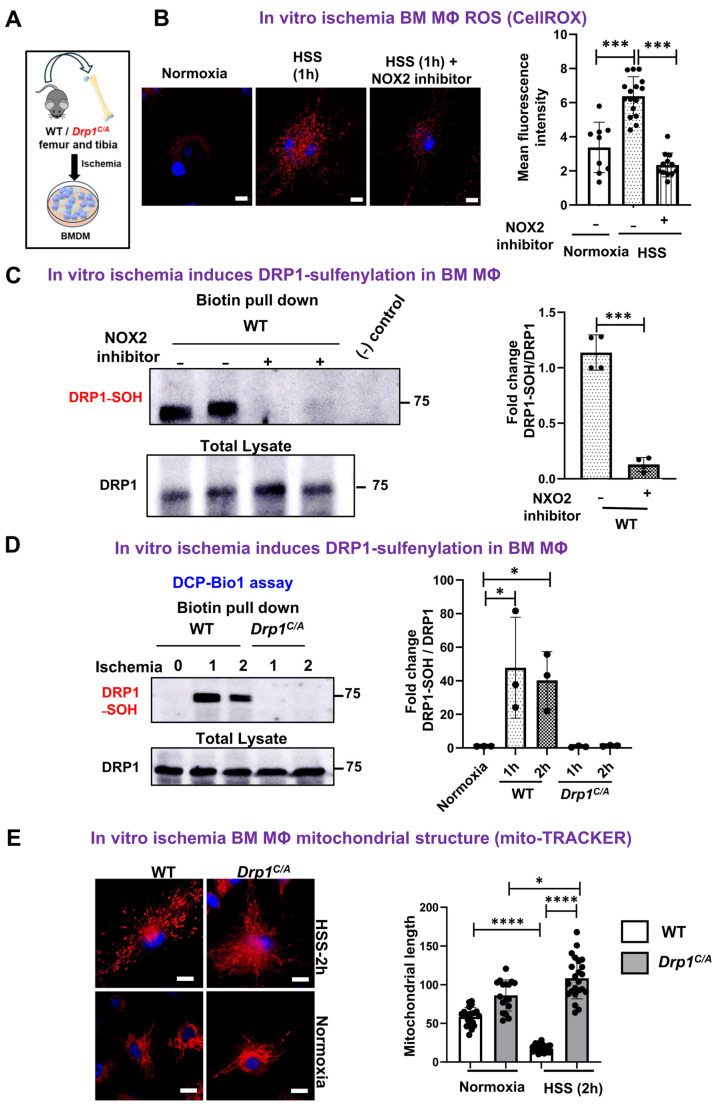
**Preventing DRP1-CysOH formation in** ***Drp1^C/A^*** **KI BMDMs during ischemia-induced mito-fusion.** (**A**) Schematic of in vitro BMDM hypoxia serum starvation (HSS) model. (**B**) IF imaging and quantification of CellROX-stained (red) WT BMDMs with or without 10 µM NOX2 inhibitor. Scale bar: 5 µM. Each dot represents number of cells quantified from n = 3 sets of BMDMs, 1-way ANOVA followed by Bonferroni’s multiple comparisons test. Immunoblotting and quantification for DRP1 sulfenylation using DCP-Bio1. (**C**) WT BMDMs with or without NOX2 inhibitor after 1 h of HSS stimulation. (**D**) WT and *Drp1^C/A^* BMDMs at indicated time post hypoxia serum starvation (HSS). DCP-Bio1 pulldown experiments were performed with a “beads-only” negative control consisting of streptavidin beads incubated with lysis buffer containing DCP-Bio1 in the absence of cell lysates. n = 3–4 sets of BMDMs, 1-way ANOVA followed by Bonferroni’s multiple comparisons test. (**E**) IF imaging of mitoTracker (red) staining and its quantification. Scale bar: 5 µM. Each dot represents number of cells quantified from n = 3 sets of BMDMs, 1-way ANOVA followed by Bonferroni’s multiple comparisons test. BMDMs from sex- and age-matched 12–18-week-old WT and *Drp1^C/A^* mice were used. Data are mean ± SEM. * *p* < 0.05, *** *p* < 0.001, **** *p* < 0.0001.

Since NOX2-derived ROS in myeloid cells play a critical role in post-ischemic reparative neovascularization [11], we investigated whether NOX2 mediates ischemia-induced DRP1 sulfenylation in macrophages using BMDMs exposed to hypoxia serum starvation (HSS), an established in vitro model of PAD [24] (Figure 4A). HSS stimulation for 1 h significantly increased ROS production in WT BMDMs, as measured by the CellROX fluorescent probe, and this response was abolished by pre-treatment with the selective NOX2 inhibitor GSK2795039 (Figure 4B). Similar results were obtained using the DCF-DA probe, where HSS-induced ROS production was suppressed by PEG-catalase (Figure S1A,B). DCP-Bio1 assay revealed that DRP1 sulfenylation (Cys-OH) was robustly induced in WT BMDMs within 1–2 h of HSS stimulation. This response was completely abolished by NOX2 inhibitor (Figure 4C) and was absent in *Drp1^C/A^* BMDMs (Figure 4D), indicating that NOX2-derived ROS are required for ischemia-induced DRP1 sulfenylation in macrophages. Previous study shows that HSS induced a transient increase in mitochondrial fission in BMDMs, peaking at 1–2 h and returning to baseline by 8 h, without changes in canonical DRP1 phosphorylation at Ser616 or Ser637, total DRP1 expression, or levels of other mitochondrial fission and fusion proteins (MFF, MFN1/2, OPA1) [24]. In contrast, *Drp1^C/A^* BMDMs displayed elongated, hyperfused mitochondria at 2 h under HSS (Figure 4E), closely resembling the phenotype observed in DRP11-deficient BMDMs [24].

**Figure 5:**
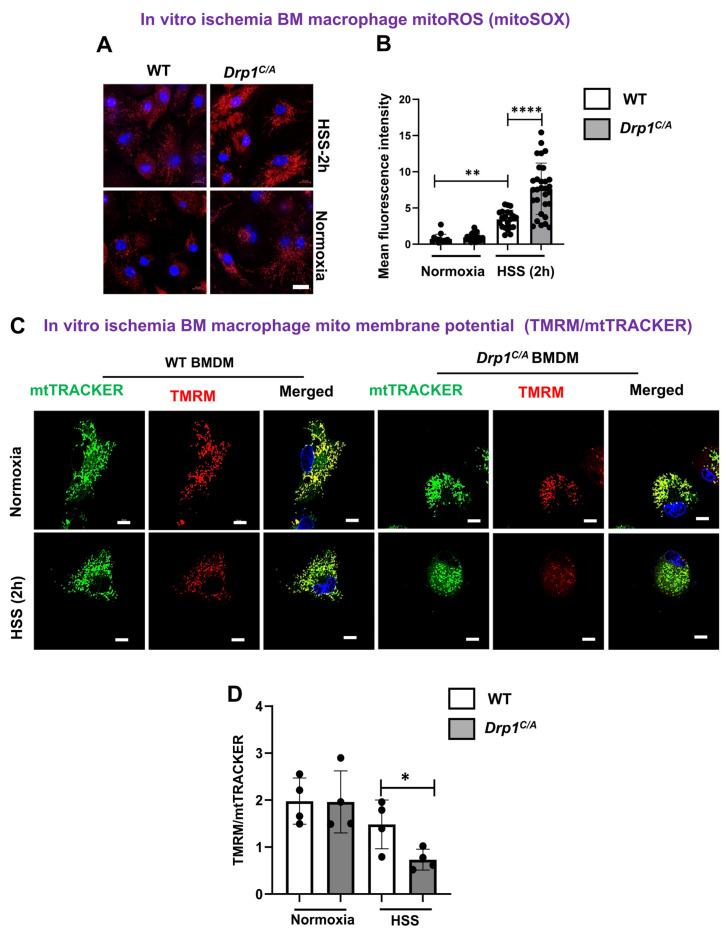
***Drp1^C/A^*** **BMDMs exhibited excess mitoROS production and reduced mito-membrane potential.** (**A**,**B**) mitoSOX staining of mitoROS (red), each dot represents number of cell quantified from n = 3 sets of BMDMs. Scale bar: 5 µM. 1-way ANOVA followed by Bonferroni’s multiple comparisons test. (**C**,**D**) TMRM (red) and mtTRACKER (green) staining and qunatification of mitochondrial membrane potentialin WT and *Drp1^C/A^* BMDMs post 2 h of HSS stimulation. Scale bar: 5 µM. n = 4 sets of BMDMs, 1-way ANOVA followed by Bonferroni’s multiple comparisons test. Data are mean ± SEM. * *p* < 0.05, ** *p* < 0.01, **** *p* < 0.0001.

We next assessed whether loss of DRP1-CysOH affects mitochondrial function by measuring oxygen consumption rate (OCR) using Seahorse analysis. Under HSS conditions, *Drp1^C/A^* BMDMs exhibited no significant differences in basal respiration, maximal respiration, or ATP production compared with WT BMDMs (Figure S2A). Consistent with these findings, no significant difference in expression of immune-responsive gene 1 (*Irg1*), which encodes the enzyme responsible for itaconate production from the TCA cycle intermediate cis-aconitate [33], was observed between WT and *Drp1^C/A^* BMDM after 8 h of HSS stimulation (Figure S2B). Despite preserved mitochondrial respiratory capacity, *Drp1^C/A^* BMDMs displayed elevated mitochondrial ROS (mitoROS) production after 2 h of HSS stimulation, as measured by MitoSOX fluorescence (Figure 5A,B). This increase in mitoROS was accompanied by a significant reduction in mitochondrial membrane potential, assessed by TMRM fluorescence, compared with WT BMDMs (Figure 5C,D). Collectively, these findings indicate that ischemia-induced DRP1 sulfenylation is dispensable for maintaining mitochondrial respiration but is required for preserving mitochondrial integrity under ischemic stress. Loss of DRP1-CysOH results in mitochondrial hyperfusion, membrane depolarization, and excessive mitoROS generation, providing a mechanistic link between redox-dependent regulation of DRP1, mitochondrial dynamics, and macrophage dysfunction in PAD.

### 3.4. Inhibition of Ischemia-Induced DRP1 Sulfenylation Suppresses AMPK Activation, Enhances Glycolysis and Skews Macrophage Polarization to Pro-Inflammatory Phenotype

**Figure 6:**
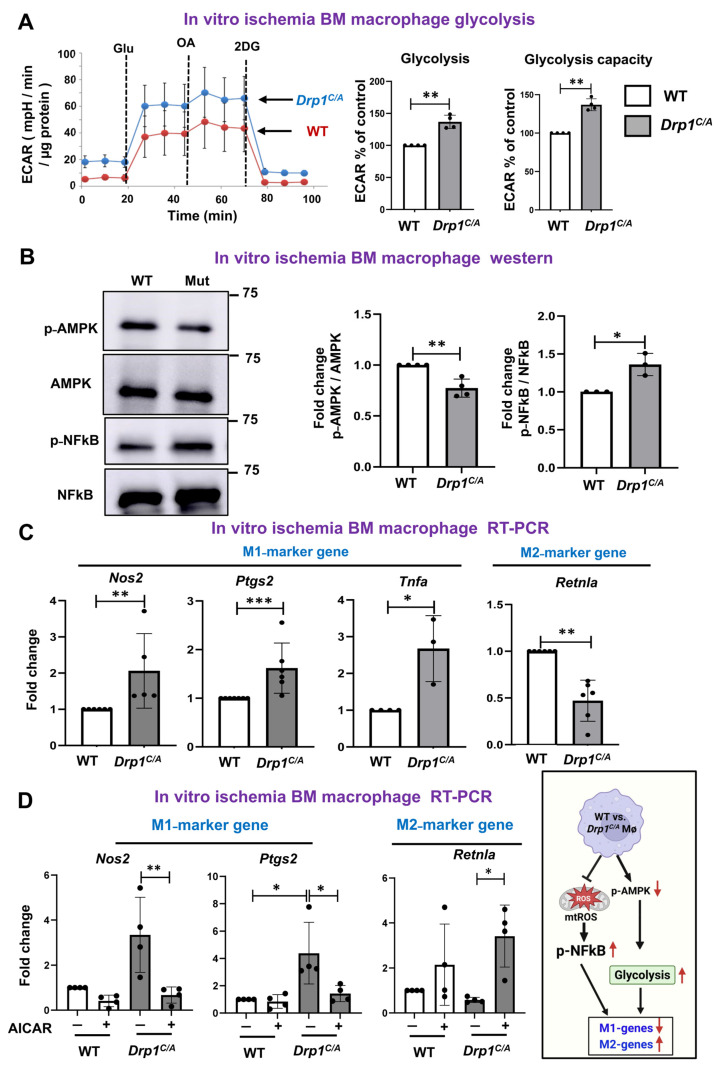
***Drp1 ^C/A^*** **BMDMs suppressed ischemia-induced p-AMPK, increased glycolysis and pro-inflammatory M1-genes, and decreased anti-inflammatory M2-genes after HSS.** WT (red) and *Drp1^C/A^* (blue) BMDMs after 2 h of HSS were used to measure (**A**) glycolysis using Seahorse analyzer, (**B**) p-AMPK, p-NFkB, total AMPK and NFkB using immunoblotting. n = 3 sets of BMDMs per group, unpaired Student’s *t*-test. (**C**) M1-and M2-marker genes normalized to *Hprt* after 8 h of HSS (**D**) with or without 100 µM AICAR pre-treatment by qRT-PCR analysis. n = 4 sets of BMDMs per group, 1-way ANOVA followed by Bonferroni’s multiple comparisons test. BMDMs from sex- and age-matched 12–18-week-old WT and *Drp1^C/A^* mice were used. Data are mean ± SEM. * *p* < 0.05, ** *p* < 0.01, *** *p* < 0.001. (Schematic hypothesis model, created in BioRender. Ushio-Fukai, M. (2026), https://BioRender.com/4zlx47i).

To directly determine the role of DRP1-CysOH in macrophage metabolic reprogramming and polarization under ischemic conditions, BMDMs were subjected to HSS in vitro. Because ischemia-driven pro-inflammatory macrophage polarization is associated with a metabolic shift from oxidative phosphorylation to aerobic glycolysis [34,35], glycolytic activity was assessed by measuring extracellular acidification rate (ECAR) using a Seahorse assay. As shown in Figure 6A, *Drp1^C/A^* BMDMs exhibited significantly increased basal glycolysis and glycolytic capacity compared with WT BMDMs under HSS. We found that there were no significant differences in the protein expression of key glycolytic enzymes including PFKFB3, GAPDH, and PKM2 between WT and *Drp1^C/A^* BMDMs following 2 h of HSS stimulation (Figure S3A). To elucidate the mechanism underlying enhanced glycolysis in *Drp1^C/A^* KI macrophages, we next examined AMPK signaling, which promotes anti-inflammatory, pro-angiogenic M2 polarization in part by suppressing glycolysis, as previously reported in *Drp1*^−/−^BMDMs [24]. Consistent with this pathway, *Drp1^C/A^* BMDMs displayed markedly reduced p-AMPK and a modest increase in NF-κB phosphorylation compared with WT controls under HSS (Figure 6B). Since we previously reported that DRP1 deficiency in BMDMs with ischemia reduces p-AMPK in a mitoROS-independent manner [24], we next examined if elevated mitoROS contributes to inflammatory signaling such as p-NFkB in *Drp1^C/A^* BMDMs under ischemia. We found that mitochondrial-targeted ROS scavenger mitoTEMPO significantly reduced p-NF-κB/NF-κB expression in *Drp1^C/A^* BMDMs with HSS stimulation (Figure S3B). These results suggest that mitoROS acts upstream of NF-κB activation in *Drp1^C/A^* BMDMs under ischemic stress conditions.

In coherence with our in vivo hindlimb ischemia findings, *Drp1^C/A^* BMDMs exposed to HSS showed significantly elevated expression of M1-associated genes (*Nos2*, *Ptgs2*, and *Tnfα*) and reduced expression of the M2-marker gene *Retnla* relative to WT BMDMs (Figure 6C). To determine whether decreased AMPK activity contributes to enhanced glycolysis and pro-inflammatory macrophage polarization in *Drp1^C/A^* BMDMs under HSS as we observed in *Drp1*^−/−^BMDMs with HSS [24], we performed rescue experiments using the AMPK activator AICAR [36,37]. Activating AMPK by AICAR at a concentration that did not affect glycolysis in WT BMDMs under HSS [24] rescued the elevated expression of pro-inflammatory M1-genes *Nos2* and *Ptgs2* in *Drp1^C/A^* BMDMs under HSS as comparable to those observed in HSS-exposed WT BMDMs (Figure 6D). Notably, AICAR also rescued the reduced expression of genes associated with the anti-inflammatory M2-marker gene in *Drp1^C/A^* BMDM under HSS (Figure 6D). These findings indicate that loss of DRP1-CysOH enhances glycolytic reprogramming and NF-κB activation while attenuating AMPK signaling, thereby skewing macrophages toward a pro-inflammatory M1-like phenotype and impairing reparative M2-like polarization under ischemic conditions in vitro.

## 4. Discussion

**Figure 7:**
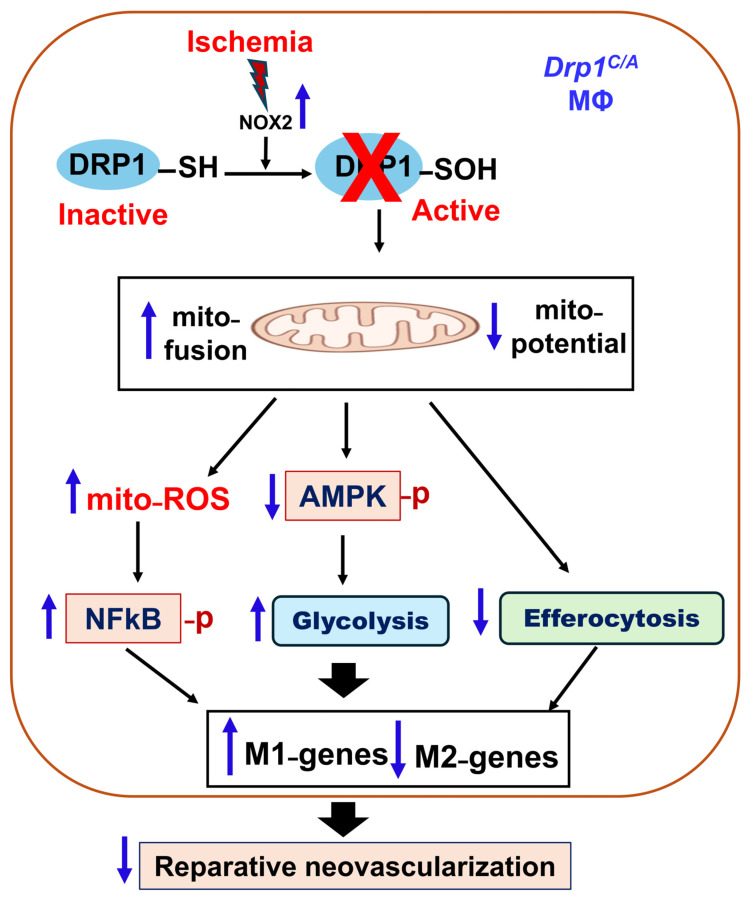
**Schematic of proposed hypothesis model.** Myeloid DRP1 sulfenylation at Cys^644^ is required for reparative neovascularization after in vivo ischemic injury. Preventing MΦ DRP1-Cys oxidation under in vitro ischemia increases glycolysis via reduced p-AMPK and efferocytosis as well as excess mitoROS-NFkB axis, driving pro-inflammatory M1-like MΦ polarization, which limits revascularization in experimental PAD. Upward arrow indicates increase and downward arrow indicates decrease.

ROS generated by BM cells in addition to endothelial cells are key signaling mediators required for ischemia-induced neovascularization in PAD [11,31,38,39]. We previously showed that ischemia activates the mitochondrial fission protein DRP1 in macrophages independently of canonical Ser616/Ser637 phosphorylation; however, the upstream regulatory mechanism remained unclear. Here, we identify ischemia-induced cysteine sulfenylation of DRP1 as a critical redox-dependent modification in BM cells, especially macrophages that are required for effective perfusion recovery and angiogenesis after HLI. BM chimeric mice expressing a “redox-dead” *Drp1^C/A^* KI mutant displayed impaired post-ischemic revascularization, accompanied by excessive neutrophil accumulation, persistent pro-inflammatory M1 macrophage polarization, and reduced M2 macrophage responses in ischemic muscle. Mechanistically, ischemic stress induced NOX2-ROS-dependent DRP1 sulfenylation and mitochondrial fission independently of changes in DRP1 phosphorylation. In contrast, disruption of DRP1 cysteine oxidation impaired mitochondrial dynamics, resulting in metabolic and inflammatory reprogramming characterized by enhanced glycolysis, increased mitoROS–NF-κB signaling, reduced AMPK activation and efferocytosis, and a shift toward a pro-inflammatory macrophage phenotype (Figure 7).

DRP1 is a central mediator of mitochondrial fission, and its activity is tightly controlled by multiple post-translational modifications (PTMs), including phosphorylation, SUMOylation, ubiquitination, S-nitrosylation, and O-GlcNAcylation, which collectively fine-tune mitochondrial and cellular dynamics [40]. We previously reported that ischemia activates DRP1 in macrophages without phosphorylation, which is required for reparative neovascularization [24]. In the present study, we demonstrate for the first time that DRP1-CysOH formation is dramatically increased in BM cells following HLI. Importantly, BM chimera mice harboring the redox-dead DRP1-C^631^A KI mutation exhibit impaired limb perfusion recovery and angiogenesis after HLI, establishing a functional requirement of DRP1 sulfenylation in BM cells for driving ischemic revascularization. Furthermore, in vitro ischemia increased mitochondrial fission, H_2_O_2_ levels and DRP1-CysOH formation in BMDMs, independently of phosphorylation at Ser616 or Ser637 [24], confirming that macrophage DRP1 is activated primarily through Cys^631^ sulfenylation rather than canonical phosphorylation pathways.

Our previous studies identified NOX2 as a major source of ROS in BM-derived cells that promotes reparative neovascularization in ischemic muscle [11,12]. Consistent with reports showing that NOX2-derived ROS facilitate monocyte-to-M2-like macrophage polarization and inflammation resolution [41,42], pharmacological inhibition of NOX2 markedly attenuated both HSS-induced ROS production and DRP1-CysOH formation in WT BMDMs. These findings suggest that NOX2-derived ROS act upstream of DRP1 cysteine oxidation in ischemic macrophages. Although potential off-target effects of the inhibitor cannot be excluded, future studies using BMDMs isolated from Nox2 deficient mice will further validate this mechanism. Collectively, our data supports a model in which NOX2-derived ROS generated during ischemia are sensed by DRP1 through Cys631 sulfenylation, thereby promoting reparative macrophage reprogramming and post-ischemic neovascularization.

Ischemia-induced revascularization requires coordinated pro-angiogenic and metabolic reprogramming of macrophages, in which the balance between oxidative metabolism and glycolysis critically influences inflammatory status and reparative function [16]. Our findings identify myeloid DRP1 sulfenylation as a previously unrecognized redox-dependent mechanism that promotes reparative macrophage reprogramming and post-ischemic neovascularization through regulation of mitochondrial dynamics. Consistent with our previous findings in *MΦDrp1*^−/−^ mice [24], *Drp1^C/A^* KI BM chimera mice exhibited increased neutrophil accumulation and reduced macrophage infiltration at day 3 after HLI, despite normal monocyte recruitment. These changes were associated with a sustained increase in pro-inflammatory CD80^+^ M1-like macrophages and a reduction in reparative CD206^+^ M2-like macrophages at both days 3 and 7 post-HLI. Although macrophage numbers recovered by day 7, the polarization defect persisted, suggesting that DRP1 sulfenylation primarily regulates macrophage functional programming rather than recruitment. Notably, the early accumulation of neutrophils in *Drp1^C/A^* KI BM chimera mice also suggests a role for DRP1 sulfenylation in promoting efferocytotic clearance of apoptotic neutrophils, leading to M2-like polarization and inflammation resolution in ischemic tissues [43,44,45]. Supporting this possibility, *Drp1^C/A^* BMDM exhibited impaired efferocytosis following in vitro ischemic stimulation. While the underlying mechanisms remain to be fully defined, previous studies have shown that DRP1-mediated mitochondrial fission is required for hypoxia-induced efferocytosis and tissue repair [45,46], and that mitochondrial signaling through AMPK axis enhances macrophage uptake of apoptotic cells and the resolution of inflammation [47]. Together, these findings suggest that impaired efferocytosis may contribute, at least in part, to the excessive neutrophil accumulation and defective reparative macrophage polarization in *Drp1^C/A^* macrophages under ischemic conditions.

Mechanistically, inhibition of DRP1 sulfenylation under ischemia impaired DRP1-dependent mitochondrial fission, resulting in mitochondrial hyperfusion and dysregulated mitochondrial homeostasis. This was associated with reduced AMPK activation, enhanced glycolysis, increased mitochondrial dysfunction and mitoROS production, augmented NF-κB signaling, and impaired efferocytic capacity, ultimately driving M1-like polarization and suppressing reparative M2-like polarization. These alterations likely contribute to the impaired neovascularization in experimental PAD. Our findings are consistent with our previous studies using myeloid-specific *Drp1^−^^/−^* mice and macrophages, which demonstrated that loss of DRP1 promotes glycolytic reprogramming through suppression of p-AMPK as well as inducing mitochondrial dysfunction and excessive mitoROS generation, leading to enhanced pro-inflammatory M1-genes and reduced anti-inflammatory M2-genes [24]. In the present study, pharmacological activation of AMPK with AICAR partially rescued the exaggerated pro-inflammatory phenotype and restored anti-inflammatory marker expression in ischemic *Drp1^C/A^* macrophages. These results support a key role for impaired AMPK signaling in mediating the effects of defective DRP1 sulfenylation. In parallel, scavenging mitoROS with mito-TEMPO attenuated p-NF-κB in *Drp1^C/A^* BMDMs, indicating that excessive mitoROS contributes to inflammatory NF-κB signaling downstream of impaired DRP1 redox regulation. However, further studies examining the contribution of NF-κB signaling, including the use of specific NF-κB inhibitors to determine whether M1 polarization can be reversed, would provide additional mechanistic insight. Notably, TMRM analysis revealed a significant reduction in mitochondrial membrane potential in *Drp1^C/A^* BMDMs despite preserved oxygen consumption, suggesting that defective DRP1 sulfenylation compromises mitochondrial quality and bioenergetic integrity before overt respiratory failure. This in turn may promote electron leakage and mitoROS production, thereby activating NF-κB-dependent inflammatory pathways. Thus, these findings support a model in which DRP1 sulfenylation functions as a redox-sensitive regulator of mitochondrial dynamics that preserves mitochondrial fitness, sustains AMPK signaling, and restrains mitoROS-NF-κB activation, thereby promoting reparative macrophage polarization during ischemic tissue repair (Figure 7).

These are several limitations of the present study. First, while our data using the NOX2 chemical inhibitor supports a role of NOX2-derived ROS for DRP1 sulfenylation in macrophage reprogramming during ischemia, the specific role of NOX2 in this response should be investigated using myeloid-specific NOX2-deficient mice. Second, the present study used CellROX and DCF-DA fluorescence probes to measure ROS which was inhibited by the NOX2 inhibitor or PEG-catalase. Future studies using genetic approaches and advanced H_2_O_2_ biosensors such as HyPer7 or roGFP2-Orp1 will help define the spatial and temporal regulation of this pathway. However, implementation of these approaches in primary BMDMs, which are difficult to transfect using plasmids and adenoviruses, would require substantial optimization. Third, while loss of DRP1 sulfenylation enhanced glycolysis, more comprehensive metabolic analyses, including isotope tracing and assessment of TCA cycle immunometabolites such as succinate and itaconate [33,48,49], are needed to further define the underlying metabolic mechanisms. Fourth, whether impaired DRP1 sulfenylation activates additional inflammatory pathways, such as the mitochondrial DNA-cGAS-STING axis, remains to be determined. Fifth, because BM transplantation reconstitutes multiple hematopoietic cell populations, contributions from immune cells other than macrophages cannot be excluded. However, the consistent defects observed in *Drp1^C/A^* BMDMs support a macrophage-intrinsic role for DRP1 sulfenylation. Sixth, although rescue experiments such as re-expression of WT DRP1 in *Drp1^C/A^* macrophages could further strengthen causality, technical challenges in transfection in primary macrophages and the lack of faithful sulfenylation-mimetic, cysteine-to-aspartate substitutions (e.g., C631D) mutants limited these approaches. Finally, whether DRP1 sulfenylation similarly regulates macrophage function in other disease settings, such as diabetic PAD or vascular aging, warrants future investigation. Together, these studies will further define the therapeutic potential of targeting DRP1 redox signaling to enhance tissue repair and revascularization.

## 5. Conclusions

In conclusion, these findings uncover DRP1 sulfenylation as a redox-sensitive molecular switch that couples ischemia-induced ROS signaling to macrophage metabolic reprogramming and reparative neovascularization. By uncoupling DRP1 activation from canonical phosphorylation and identifying sulfenylation as the dominant regulatory mode in ischemic macrophages, these findings redefine how macrophage mitochondrial dynamics are controlled during tissue repair. Targeting DRP1 redox signaling may therefore represent a novel therapeutic strategy to enhance macrophage-mediated revascularization while limiting chronic inflammation in PAD.

## Data Availability

The data presented in this study are available in the article and Supplementary Materials.

## Supplementary Materials

The following supporting information can be downloaded at https://www.mdpi.com/article/10.3390/antiox15060768/s1. **Figure S1: A.** ***In vitro*** **ischemia induced H_2_O_2_ in WT BMDM.** DCF-DA (H_2_O_2_production) fluorescence in WT BMDMs during normoxia vs 1h of HSS stimulation with or without PEG-catalase. **Figure S2: Mitochondrial respiration in** ***Drp1^C/A^*** **BMDMs with ischemia is unaffected. (A).** WT and *Drp1^C/A^* BMDMs after 2 h of HSS were used to measure mitochondrial respiration rate (OCR) using Seahorse. n = 4 sets of BMDM per group. **(B)** RT-PCR of *Irg1* gene normalized to *Hprt* after 8 h of HSS. n = 4 sets of BMDM per group. BMDMs from Sex- and age-matched 12-18 weeks WT and *Drp1^C/A^* mice were used. Data are mean ± SEM. **Figure S3: mitoTEMPO inhibits excess NFΚB activation in** ***Drp1^C/A^*** **BMDM** **upon HSS.** WT and *Drp1^C/A^* BMDM after 2 h of HSS (**A**) glycolytic enzymes PFKB3, GAPDH and PKM2 **(B)** p-NFkB, and NFkB with or without 50µM mitoTMEPO pre-treatment using immunoblotting. n = 4–5 sets of BMDM per group, 1-way ANOVA followed by Bonferroni’s multiple comparisons test. BMDMs from Sex- and age-matched 12–18 weeks WT and *Drp1^C/A^* mice were used. Data are mean ± SEM. * *p* < 0.05, ** *p* < 0.01, *** *p* < 0.001. **Table S1: Antibodies used for flow cytometry based immunophenotyping**. **Table S2: Sequences of primers used for RT-PCR**.
