# Supplementary material for: Myeloid DRP1 Sulfenylation Drives Reparative Macrophage Polarization and Neovascularization in Ischemic Muscle
Source: Antioxidants (Basel). 2026 Jun 19;15(6):768. doi: 10.3390/antiox15060768 (PMC13295537; doi:10.3390/antiox15060768)
Supplement: Supplementary file 1 [file antioxidants-15-00768-s001.zip › antioxidants-4184740-supplementary.pdf]

# Supplementary Figure S1

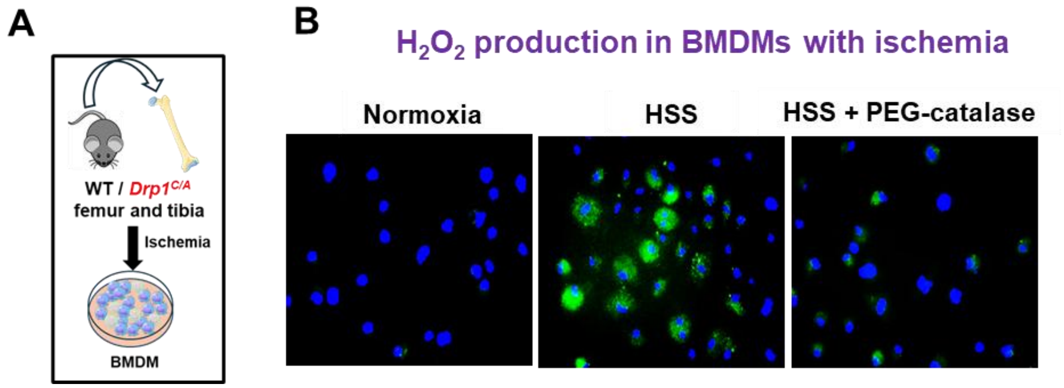


**Figure S1: A. *In vitro* ischemia induced $H_2O_2$ in WT BMDM.** DCF-DA ($H_2O_2$production) fluorescence in WT BMDMs during normoxia vs 1h of HSS stimulation with or without PEG-catalase.

## Supplementary Figure S2

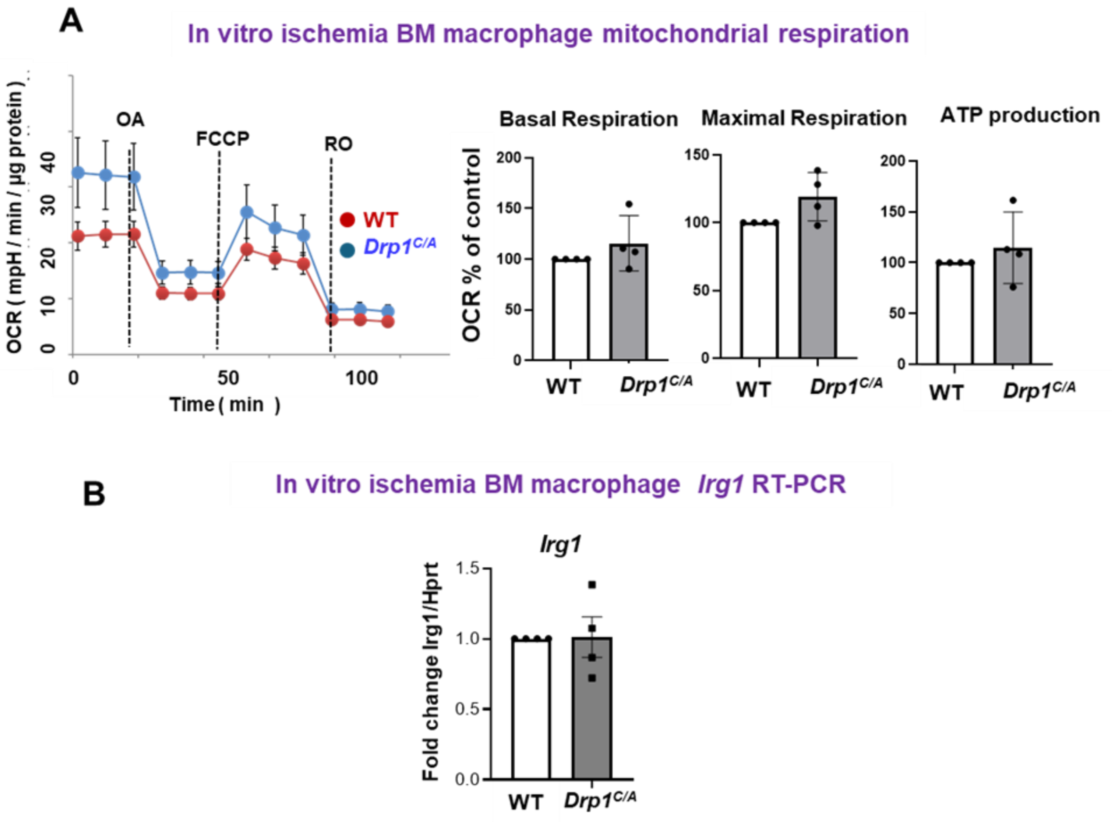


**Figure S2: Mitochondrial respiration in *Drp1$^{C/A}$* BMDMs with ischemia is unaffected. (A).** WT and *Drp1$^{C/A}$* BMDMs after 2 h of HSS were used to measure mitochondrial respiration rate (OCR) using Seahorse. n=4 sets of BMDM per group. **(B)** RT-PCR of *Irg1* gene normalized to *Hprt*after8 h of HSS. n=4 sets of BMDM per group. BMDMs from Sex- and age-matched 12-18 weeks WT and Drp1C/A mice were used. Data are mean ± SEM.

## Supplementary Figure S3

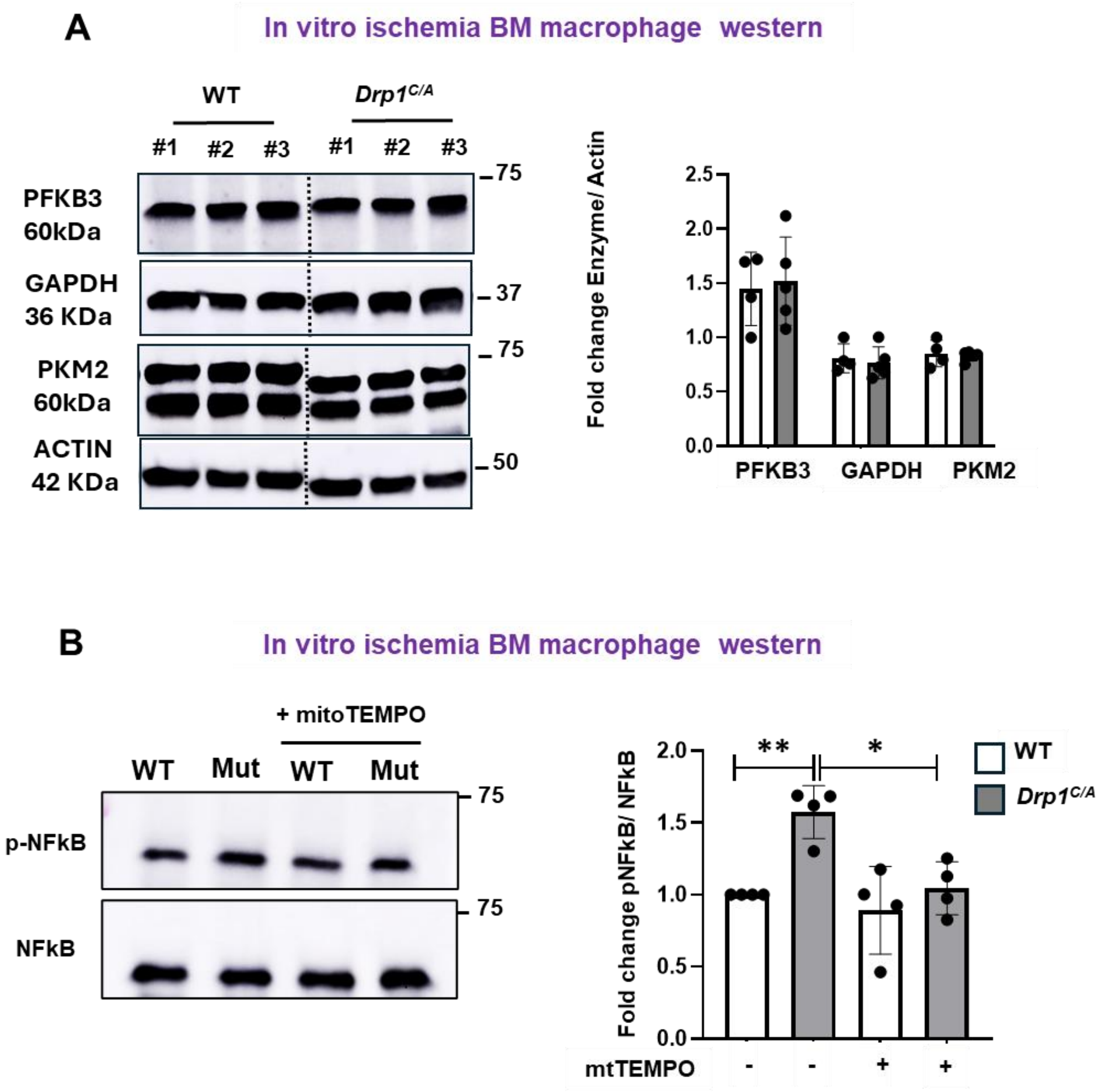


**Figure S3: mitoTEMPO inhibits excess NFKB activation in *Drp1*$^{C/A}$ BMDM upon HSS.** WT and *Drp1*$^{C/A}$ BMDM after 2 h of HSS (**A**) glycolytic enzymes PFKB3, GAPDH and PKM2 **(B)** p-NFkB, and NFkB with or without 50μM mitoTMEPO pre-treatment using immunoblotting. n=4-5 sets of BMDM per group, 1-way ANOVA followed by Bonferroni's multiple comparisons test. BMDMs from Sex- and age-matched 12-18 weeks WT and *Drp1*$^{C/A}$ mice were used. Data are mean ± SEM. *P<0.05, **P<0.01, ***P<0.001.

**Table S1: Antibodies used for flow cytometry based immunophenotyping**

| Antibody | Source | Catalog No. |
|---|---|---|
| PE anti mouse CD45 | Biolegend | 147712 |
| PE/Cyanine anti mouse CD11b | Biolegend | 101216 |
| PE anti mouse CD11b | Invitrogen | 12-0112-82 |
| FITC anti-mouse Ly-6C | BD Bioscience | 553104 |
| Alexa 647 anti mouse Ly-6G | Biolegend | 127610 |
| PE/Cyanine anti mouse Ly-6G | Biolegend | 127624 |
| PerCP-Cyanine5.5 anti mouse F4/80 | Invitrogen | 45-4801-80 |
| APC anti mouse F4/80 | Biolegend | 123116 |
| BV421 anti mouse CD80 | BD Bioscience | 562611 |
| BV605 anti mouse CD206 | Biolgend | 141721 |
| PerCP-Cyanin5.5 Rat IgG2a kappa isotype control | Invitrogen | 45-4321-80 |
| APC Mouse IgG1, K isotype control | BioLegend | 400119 |
| BV421 Hamster IgG2, K Isotype control | BioLegend | 562612 |
| Brilliant Violet 605, Rat IgG2a, κ Isotype Control | BioLegend | 400540 |

**Table S2: Sequences of primers used for RT-PCR**

| Gene | Forward | Reverse |
|---|---|---|
| *Nos2* | TGAAGAAAACCCCTTGTGCT | TTCTGTGCTGTCCCAGTGAG |
| *Ptgs2* | ACTGGGCCATGGAGTGGACTTAAA | AACTGCAGGTTCTCAGGGATGTGA |
| *Retnla* | CCCTGCTGGGATGACTGCTA | TGCAAGTATCTCCACTCTGGATCT |
| *Mertk* | CAGGGCCTTTACCAGGGAGA | TGTGTGCTGGATGTGATCTTC |
| *Gpnb* | GGGCCATGAACAGTATCCCG | CCTTCTGGCATCTGGGGAAC |
| *Hprt* | CTGGTGAAAAGGACCTCTCGAAG | CCAGTTTCACTAATGACACAAACG |
